# Detection of toxic choline based on Mn_2_O_3_/NiO nanomaterials by an electrochemical method

**DOI:** 10.1039/c9ra07459g

**Published:** 2019-10-31

**Authors:** Mohammed M. Rahman, M. M. Alam, Abdullah M. Asiri

**Affiliations:** Chemistry Department, Faculty of Science, King Abdulaziz University Jeddah 21589 P.O. Box 80203 Saudi Arabia; Department of Chemical Engineering and Polymer Science, Shahjalal University of Science and Technology Sylhet 3100 Bangladesh alam-mahmud@hotmail.com +966567697830

## Abstract

In this study, a novel *in situ* choline sensor was assembled by attaching the binary Mn_2_O_3_/NiO nanoparticles (NPs) onto a glassy carbon electrode (GCE). Initially, Mn_2_O_3_/NiO NPs were synthesized *via* a wet-chemical process and fully characterized *via* XRD, XPS, FESEM, EDS, FTIR and UV-Vis methods. The analytical performances of the choline sensor were evaluated by an electrochemical method in the phosphate buffer phase. The estimated linear dynamic range (LDR) was found to be 0.1 nM to 0.1 mM. The other analytical performances of the choline sensor, such as sensitivity (16.4557 μA μM^−1^ cm^−2^) and detection limit (5.77 ± 0.29 pM), were also calculated very carefully from the calibration plot. Overall, the choline sensor exhibited a reliable reproducibility, *in situ* validity, selectivity, interference effect, stability, and intra-day and inter-day performances with high accuracy in a short response time. Moreover, the probe was successfully applied to detect choline in real human, mouse and rabbit serum. This fabrication route would be a novel approach for the detection of selective biochemical sensor in the healthcare and biomedical fields.

## Abstract 2

In this study, a novel *in situ* choline sensor was assembled by attached the binary Mn_2_O_3_/NiO nanoparticles onto glassy carbon electrode, which might be a reliable way to develop of future sensor in the field of biomedical and healthcare fields.

## Graphical abstract

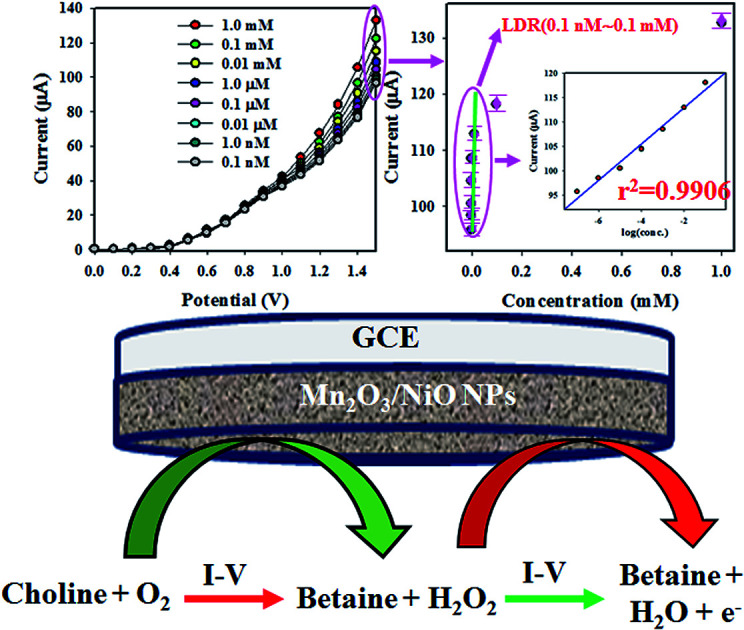

## Introduction

Choline is one of the essential nutrients for humans, and it is obtained from the liver of beef and chicken, soybeans, wheat germ and eggs.^1,2^ To ensure good public health, the recommended choline amounts are 550.0 mg day^−1^ for men and 425 mg day^−1^ for women.^3^ Choline executes a number of essential functions in the human brain and acts as a precursor for the synthesis of phospholipids. It also facilities the transport of cholesterol in human body.^4^ The critical neurotransmitter acetylcholine, which mediates memory storage in human brain, is generated from choline.^5^ Besides, choline is a primary methyl group donor to the cellular methylation reaction in the human biological system.^6^ Therefore, the deficiency of choline in human body may cause various health effects, such as fatty liver development, damage of liver and muscles, and enhanced sensitivity of carcinogens.^7–11^ It has been reported that the abnormal metabolism of choline might cause neurodegenerative disorders, such as Parkinson's and Alzheimer's diseases. It also increases the risk of prostate cancer.^12^ The source of choline for infants is only milk; hence, a supplement of choline is necessary. It is well-known that choline is very important for the human biological system; therefore, it is essential to develop a reliable system to detect choline in human serum as well as in baby food (milk). The enzymatic detection of choline using thermal, colorimetric and electrochemical methods has been reported.^13–16^ Among the choline detection methods, the electrochemical process is more reliable.

The transition metal oxide, Mn_2_O_3_, has a high band gap energy of 4.2 eV, which allows Mn_2_O_3_ to be applied in various fields of application, such as photo-catalysts, electrochemical sensors, supercapacitors, and lithium ion batteries.^17–19^ Therefore, Mn_2_O_3_ has been successfully implemented as an electrochemical sensor to detect acetone,^20^ 3,4-diaminotoluene,^21^ hydrazine,^22^*etc.* Due to the attractive electrochemical properties of NiO, it has been applied in a number of electrochemical sensor applications, such as 2,4-dinitrophenol,^23^ 4-aminophenol,^24^ and 4-methoxyphenol.^24^ Thus, the goal of this study is to develop a sensor based on Mn_2_O_3_/NiO NPs, since Mn_2_O_3_ and NiO have performed successfully as electrochemical sensors using the *I*–*V* method. The combination of Mn_2_O_3_ and NiO transition metal oxides might work as a bio-sensor in electrochemical approaches.

Here, Mn_2_O_3_/NiO nanoparticles have involved a great deal of consideration due to their chemical, structural, physical, and optical properties in terms of large-active surface area, high-stability, high porosity, and permeability, which directly depend on the structural morphology of the reactant precursors (nickel chloride and manganese sulphate) in the formation of Mn_2_O_3_/NiO nanoparticles in a basic medium at a low-temperature. Mn_2_O_3_/NiO nanoparticles were synthesized by a wet-chemical method using NaOH as a reducing agent at ambient conditions. This technique has several advantages, including facile preparation, accurate control of the reactant temperature, ease of handling, one-step reaction, and high-porosity as well as the porous nature. The optical, morphological, electrical, and chemical properties of Mn_2_O_3_/NiO nanomaterials are of huge significance from the scientific aspect, compared to other undoped materials. Non-stoichiometry, mostly oxygen vacancies, results in the conducting nature in doped nanomaterials. The formation energy of oxygen vacancies and metal interstitials in a semiconductor is very low, and thus, these defects are readily formed, resulting in the experimentally elevated conductivity of Mn_2_O_3_/NiO nanoparticles compared to other undoped materials. Mn_2_O_3_/NiO nanoparticles have also attracted considerable interest due to their potential applications in the fabrication of opto-electronics, electro-analytical devices, selective detection of assays, sensor devices, hybrid-composites, electron-field emission sources, biochemical detection, and surface-enhanced Raman properties *etc.* Mn_2_O_3_/NiO nanoparticles offer improved performance due to the large-active surface area, which increases the conductivity and current responses of the Mn_2_O_3_/NiO NP/Nafion/GCE assembly during an electrochemical investigation.

In this study, a selective and sensitive chemical sensor was fabricated based on Mn_2_O_3_/NiO NPs. Selective choline performances were totally optimized very carefully in terms of analytical parameters. The electrochemical sensor was found to be selective towards choline and the detailed analytical performances of this choline sensor, such as sensitivity, linear dynamic range, detection limit, response time, reproducibility, stability, and interference effect were evaluated. Based on the obtained electrochemical results, the selective choline sensor exhibited reliable and satisfactory results. Therefore, this research might introduce a way to develop a sensor based on an electrochemical method in the fields of bio-medical and healthcare sectors at broad scales.

## Experimental section

### Materials and methods

To synthesize Mn_2_O_3_/NiO NPs, analytical grade chemicals, such as NiCl_2_·6H_2_O and MnSO_4_·H_2_O, were purchased and used directly as received from the supplier. As supplementary chemicals of the research study, a number of bio-chemicals, such as folic acid, creatine, d-glucose, ascorbic acid, choline, l-glutamic acid, l(+)-lactic acid, cholesterol, and l(+)-aspartic acid were obtained from Sigma-Aldrich (USA). Beside, NH_4_OH, Nafion (5% Nafion suspension in ethanol) and monosodium and disodium phosphate buffers were used to execute this research work. Thermo-scientific K-α1 1066 X-ray photoelectron spectroscopy (XPS) with an excitation radiation source (A1 Kα1, beam spot size = 300.0 μm, pass energy = 200.0 eV, pressure ∼ 10–8 torr) was implemented to study the binding energies and oxidation states of the elements present in the as-synthesized Mn_2_O_3_/NiO NPs. To characterize the absorption of visible light, the as-prepared NPs were examined using a thermos-scientific 300 UV-Vis spectrometer, while a thermos-scientific NICOLET iS50 (Madison, WI, USA) FTIR spectrometer was used to study the existing functional groups in Mn_2_O_3_/NiO NPs. The elemental composition and structural morphology were inspected by FESEM analysis equipped with EDS. The crystallinity and the particle size of the as-prepared NPs were evaluated by powder X-ray diffraction (XRD) using an ARL™ X'TRA powder diffractometer. The electrochemical (*I*–*V*) investigation was performed using a Keithley electrometer (6517A, USA). Real samples were collected from a local medical center. All experiments were performed in compliance with relevant laws or institutional guidelines (CEAMR, King Abdulaziz University). After the dilution of collected serum and urine samples in the PBS buffer, they were analyzed with the fabricated Mn_2_O_3_/NiO NP sensor by an electrochemical method at room conditions. All experiments were performed in compliance with the relevant laws and institutional guidelines (Center of Excellence for Advanced Material Research at King Abdulaziz University, Jeddah, Saudi Arabia). All animal procedures were performed in accordance with the Guidelines for Care and Use of Laboratory Animals of “Center of Excellence for Advanced Materials Research (CEAMR)” and approved by the Animal Ethics Committee of “King Abdul Aziz University”.

### Synthesis of Mn_2_O_3_/NiO NPs

0.1 M solutions of NiCl_2_·6H_2_O and MnSO_4_·H_2_O were prepared in 100.0 mL round bottom flasks using de-ionized water. A 250.0 mL of conical flask was taken and 50.0 mL of each prepared solution were added. Then, the conical flask was placed on a hot plat at 80 °C temperature with continuous magnetic staring. To gradually increase the pH of the resultant solution, a 0.1 M prepared NH_4_OH solution was slowly added dropwise. As the pH of the solution was increased, the metals started to co-precipitate in the form of metal hydroxide to form nuclei of crystal formation. As the pH was continuously increased, the metal hydroxides were precipitated and got adsorbed on the nuclei of the crystals to form large crystallites. At pH 10.5, all metals were precipitated quantitatively as crystals of metal hydroxides. The proposed reactions are presented as follows (eqn (i)–(iv)):

i NH_4_OH_(l)_ ⇆ NH_4(aq)_^+^ + OH_(aq)_^−^

ii NiCl_2(s)_ → Ni_(aq)_^2+^ + 2Cl_(aq)_^−^

iii MnSO_4(s)_ → Mn_(aq)_^2+^ + SO_4(aq)_^2−^

iv Ni_(aq)_^2+^ + Mn_(aq)_^2+^ + OH_(aq)_^−^ + *n*H_2_O ⇆ Mn(OH)_2_·Ni(OH)_2(s)_·*n*H_2_O↓

The resultant precipitate was separated from the aqueous medium and washed with acetone and de-ionized water, consecutively. The washed crystals of metal hydroxides were allowed to dry at a 110 °C temperature inside an oven overnight. A similar metal hydroxide formation has been described by previous authors.^25–27^ After that, the nano-crystals of metal hydroxides were subjected to calcination in a muffle furnace at a 500 °C temperature for 6 h. In this calcination process, metal hydroxides were oxidized in the presence of atmospheric oxygen and got converted to their oxide form with a higher oxidation number. The corresponding oxidation reaction (eqn (v)) in the muffle furnace is shown below.

v Mn(OH)_2_·Ni(OH)_2(s)_ + O_2_ → Mn_2_O_3_·NiO + H_2_O

### Fabrication of GCE with Mn_2_O_3_/NiO NPs

The working electrode of the desired bio-sensor was fabricated with Mn_2_O_3_/NiO NPs. To do this, slurry of Mn_2_O_3_/NiO NPs was prepared using ethanol, which was deposited onto a GCE as a very thin layer. Subsequently, the modified GCE was allowed to dry at ambient room conditions. Since this sensor will be used to detect in the aqueous medium, the stability of the fabricated working electrode is necessary. Therefore, the binding strength between NPs and the GCE was enhanced by the addition of a drop of Nafion (5% Nafion suspension in ethanol) onto the modified dry GCE, followed by putting inside an oven at a 35 °C temperature for an adequate time to dry up the assembled working electrode completely. Thus, Mn_2_O_3_/NiO NP/binder/GCE was applied to detect choline in an optimized buffer system. The desired choline bio-sensor was assembled by an electrometer, where the recently fabricated electrode acted as a working electrode, while a Pt-wire acted as the counter electrode. To execute the analytical performances of the projected choline bio-sensor, choline solutions based on various concentrations ranging from 1.0 mM to 0.1 nM were prepared with de-ionized water and used as the target analyte. A calibration curve was plotted as the concentration of choline *vs.* current, and the linearity (*r*^2^) of this resulted calibration curve was estimated. Therefore, depending on the maximum linearity of the calibration plot, the linear dynamic range (LDR) was computed. The sensitivity and detection limit (DL) of the projected choline bio-sensor were estimated from the slope of the calibration curve. It should be mentioned that, the assembled electrochemical sensor was a simple two electrode system. During the electrochemical investigation by the assembled choline bio-sensor, the buffer solution in the detecting beaker (10.0 mL) was taken as a constant throughout the whole experimentation.

## Result and discussions

### XPS analysis of Mn_2_O_3_/NiO NPs

**Fig. 1:**
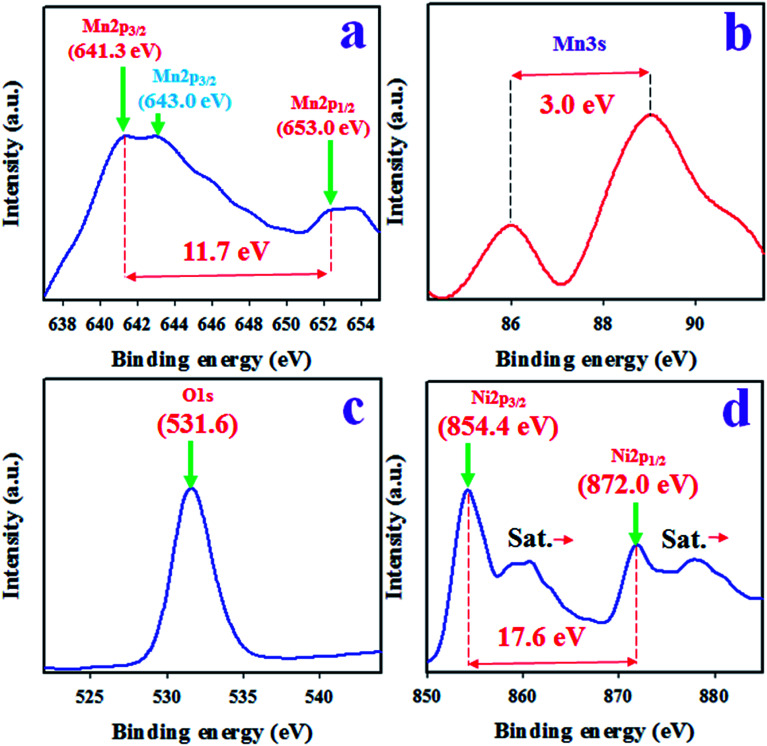
The XPS analysis of sample Mn_2_O_3_/NiO NPs. (a) Core-level XPS of Mn2p, (b) Mn3s orbit, (c) O1s orbit, and (d) high resolution XPS of Ni2p level.

To evaluate the surface composition and valence states of the as-synthesized Mn_2_O_3_/NiO NPs, X-ray photoelectron spectroscopy (XPS) analysis was performed, as presented in Fig. 1. Based on the XPS spectrum in Fig. 1, Mn2p, Mn3s, O1s and Ni2p spin orbits are observed. The XPS spectrum of Mn2p shows two identical high resolution peaks at the binding energies of 641.3 and 653.0 eV, corresponding to Mn2p_3/2_ and Mn2p_1/2_. The spin energy separation of Mn2p orbits is equal to 11.7 eV as represented in Fig. 1(a) and it indicates the oxidation state of Mn^4+^ in Mn_2_O_3_/NiO NPs.^28,29^ Another Mn2p_3/2_ peak is centered at 643.0 eV. To conclude the oxidation state, Mn3s orbit is evaluated as in Fig. 1(b) and the spin energy separation of Mn3s doublet is 3.0 eV. Therefore, it is confirmed that the oxidation state of Mn^3+^ is in the sample of Mn_2_O_3_/NiO NPs.^30^ As it is illustrated in Fig. 1(c), the peak of O1s at 531.6 eV can be assigned to the O^2−^ oxidation state in the Ni–O bond.^31,32^ Furthermore, the Fig. 1(d) represents the XPS spectrum of Ni2p, which exhibits two peaks at 854.4 and 872.0 eV, corresponding to Ni2p_3/2_ and Ni2p_1/2_ spin orbitals, with a spin energy separation of 17.6 eV, a characteristic value of the Ni^2+^ oxidation state in NiO.^33,34^

### Optical and structural analysis of Mn_2_O_3_/NiO NPs

**Fig. 2:**
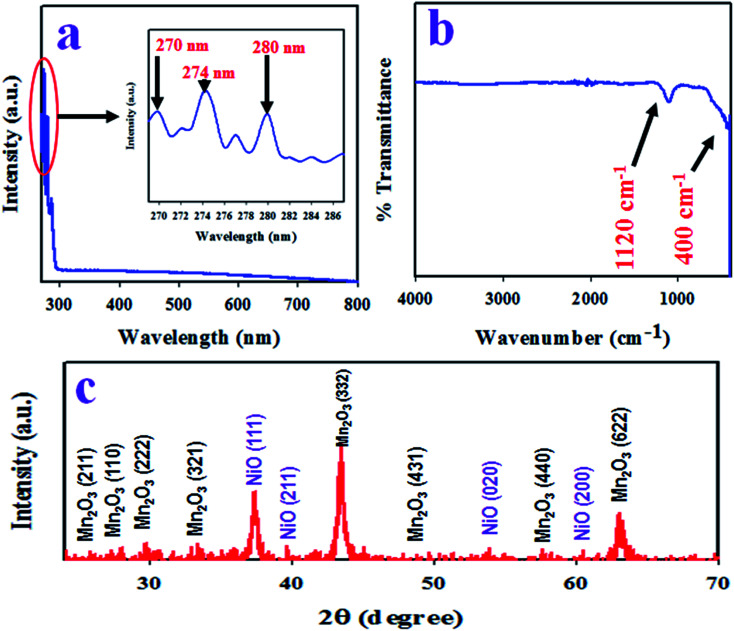
Optical and structural analysis of the as-synthesized Mn_2_O_3_/NiO NPs. (a) UV-Vis spectrum, (b) FTIR, and (c) X-ray diffraction spectrum of Mn_2_O_3_/NiO NPs.

To identify the absorption of visible light, the as-synthesized Mn_2_O_3_/NiO NPs were analyzed by UV-Vis spectroscopy in the range of 265 nm to 800 nm. The resulted UV-Vis spectrum is illustrated in Fig. 2(a). As it is observed from Fig. 2(a), the three absorption bands at 270, 274 and 280 nm wavelengths correspond to associate band gap energies of 4.59, 4.53 and 4.43 eV. The three absorption bands in the range of 265 to 800 nm wavelengths are assigned to the transition of Ni^2+^ in Mn_2_O_3_/NiO NPs.^35,36^

To identify the functional groups existing in the as-prepared Mn_2_O_3_/NiO NPs, FTIR investigation was executed in the wavenumber range of 400 to 4000 cm^−1^. The obtained FTIR spectrum is demonstrated in Fig. 2(b) and two major peaks at 400 and 1120 cm^−1^ are observed. The peak at the 400 cm^−1^ wavenumber can be ascribed to the Mn–O vibration and the peak at 1120 cm^−1^ is associated to the bending mode of OH^−^.^37,38^

The resulted X-ray diffraction spectrum of Mn_2_O_3_/NiO NPs is shown in Fig. 2(c) and the crystalline planes of Mn_2_O_3_ are (211), (222), (321), (332), (431), (440), and (622). In comparison to the standard XRD data of pure Mn_2_O_3_, a great similarity was found with JCPDS no. 041-1442 and the data reported by previous authors.^39,40^ Two major crystalline planes of NiO, namely (111) and (200), were found in the XRD pattern in Fig. 2(c), which has a great similarity to the standard data of pure NiO JCPDS no. 047-1049 and the data reported in articles.^41,42^ Using the Debye–Scherrer's formula given in eqn (vi), the particle size of NPs was calculated and it was found to be 17.87 nm at peak Mn_2_O_3_ (332).

vi *D* = 0.9*λ*/(*β* cos *θ*)

Here, *λ* is the wavelength of X-ray radiation (1.5418 Å), *β* is the full width at half (FWHM) of the peak at a diffracted angle *θ*.

### Morphology and elemental analysis of the as-prepared NPs

**Fig. 3:**
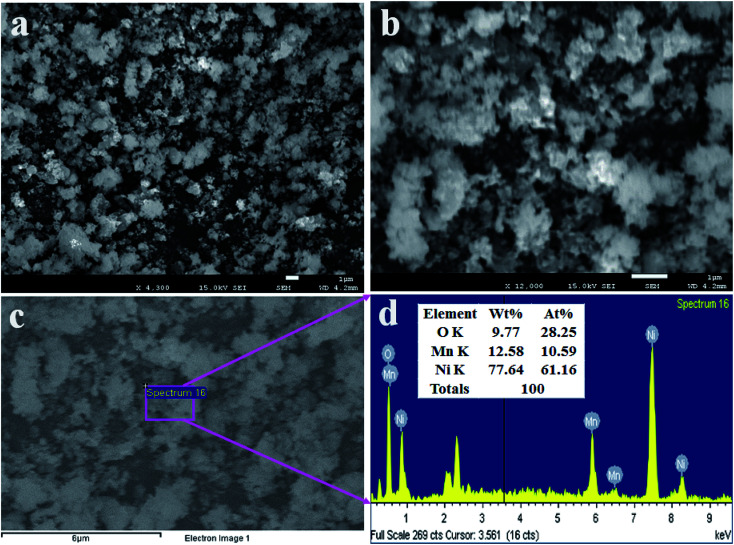
Morphological and elemental analyses of NPs. (a and b) Low- and high-magnified FESEM images of Mn_2_O_3_/NiO NPs and (c and d) the EDS analysis report.

To analyze the structural morphology of Mn_2_O_3_/NiO NPs, FESEM was performed as presented in Fig. 3(a and b). As it is observed from Fig. 3(a and b), the as-synthesized nanomaterials are nanoparticles in shape with an irregular arrangement. This similar observation was obtained from the EDS report, as shown in Fig. 3(c). The elemental compositions of Mn_2_O_3_/NiO NPs are demonstrated in the EDS analysis report, as shown in Fig. 3(d). As it is analyzed by EDS, the elemental compositions are O 9.77%, Mn 12.58% and Ni 77.64% as the weight percent.

### Applications: *in situ* choline sensor development

**Fig. 4:**
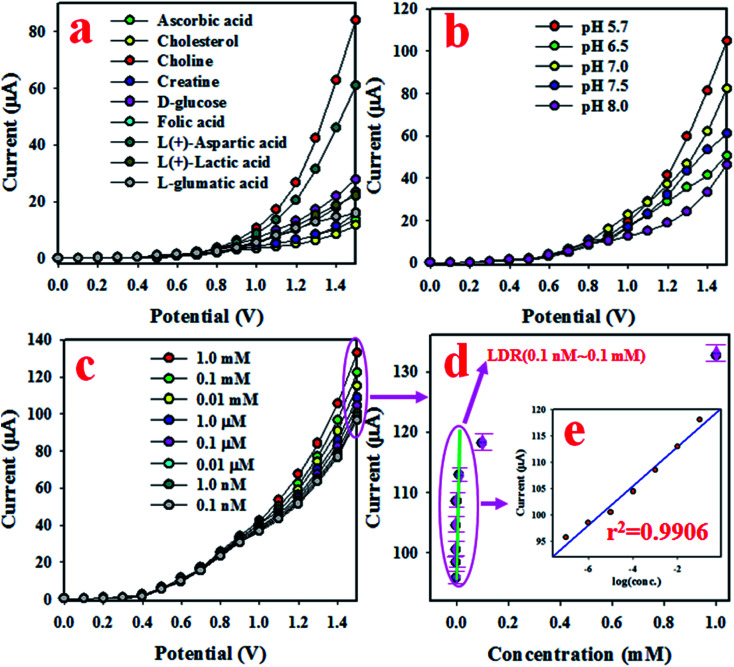
Optimization and analytical performances of the choline sensor based on the Mn_2_O_3_/NiO NP/binder/GCE. (a) Selectivity, (b) pH optimization, (c) electrochemical responses based on the concentration variation of choline from lower to higher, and (d) the calibration curve [inset current *vs.* log(conc.)].

In this approach, an electrochemical sensitive sensor was fabricated using a GCE coated with Mn_2_O_3_/NiO NPs with an Nafion conducting binder, which was used to selectively detect choline in the phosphate buffer medium. At an early stage of the optimization of the as-fabricated sensor based on Mn_2_O_3_/NiO NPs/binder/GCE, the fabricated sensor was analyzed to test the selectivity. To execute the sensor performance, several bio-chemical samples at micro-level concentrations, such as folic acid, creatine, d-glucose, ascorbic acid, choline, l-glutamic acid, l(+)-lactic acid, cholesterol, and l(+)-aspartic acid, were analyzed in the potential range of 0 to +1.5 V with a 0.1 M phosphate buffer system, and are presented in Fig. 4(a). As it is observed from Fig. 4(a), choline was found to exhibit the highest electrochemical response with the Mn_2_O_3_/NiO NP/binder/GCE fabricated sensor probe. The fabricated sensor is very active and efficient in the presence of choline compared to other biological substances. Here, the electrochemical detection process of selective choline based on the Mn_2_O_3_/NiO NP/binder/GCE responded significantly well. Choline and dissolved oxygen molecules are adsorbed onto the surface of the working electrode in the electrochemical method (with respect to the applied potential), and betaine and hydrogen peroxide are produced. Subsequently, the resulting H_2_O_2_ was automatically converted into H_2_O and released e^−^. These released electrons significantly enhanced the resultant current in the electrochemical process compared to other bio-chemicals. Furthermore, to get the maximum electrochemical responses, the fabricated sensor was tested in various phosphate buffer media. The performance was analyzed at a 0.01 μM concentration of choline with an applied potential of 0 to +1.5 V, as shown in Fig. 4(b). Obviously, the choline sensor based on Mn_2_O_3_/NiO NP/binder/GCE shows the maximum electrochemical response in a phosphate buffer system with pH 5.7.

Fig. 4(c) presents the electrochemical response of choline concentration ranging from 1.0 mM to 0.1 nM, and it can be observed that the electrochemical responses are completely distinguishable from lower to higher concentrations. This similar tendency of electrochemical responses has been reported previously to measure various toxins.^43–46^ The current data from Fig. 4(c) at an applied potential of +1.5 V have been collected and presented as current *vs.* concentration of choline in Fig. 4(d), known as the calibration curve. The slope of the calibration curve was measured and was used to calculate the sensitivity of the choline biosensor, which was equal to 16.4557 μA μM^−1^ cm^−2^, a value that is appreciable. At a signal to noise ratio of 3, the slope of the calibration curve was also used to measure the detection limit (DL) of the choline bio-sensor based on Mn_2_O_3_/NiO NP/binder/GCE and it was found to be 5.77 ± 0.29 pM, a result that might be considerably lower. To estimate the linearity of the calibration plot, the current data are plotted against the concentration of choline in logarithmic scale, as shown in Fig. 4(d) and inset Fig. 4(e). As it is observed from Fig. 4(e), the current data are fitted with a regression coefficient (*r*^2^) value of 0.9906 in the concentration range of 0.1 nM to 0.1 mM, which is identified as the linear dynamic range (LDR). Obviously, the LDR was found to be a very wide range of concentrations.

**Fig. 5:**
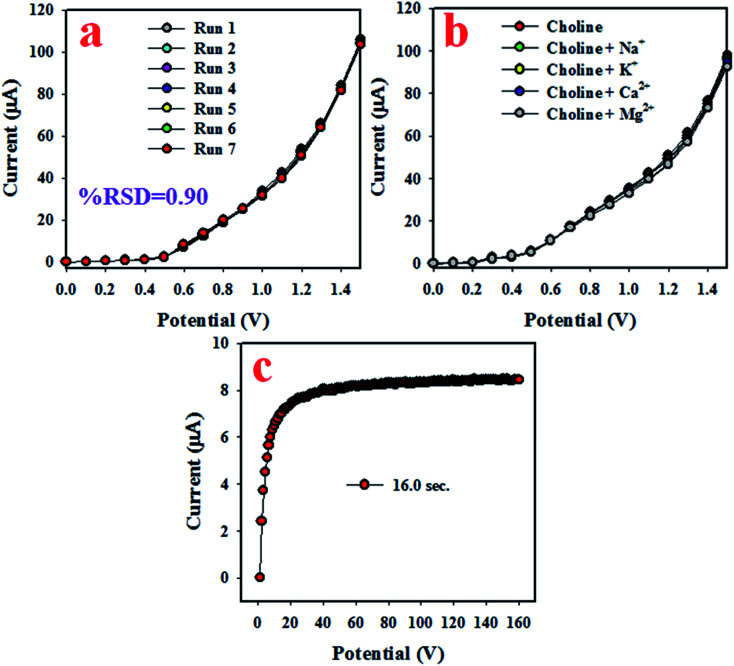
The reliability performances of the choline sensor based on Mn_2_O_3_/NiO NP/binder/GCE. (a) Reproducibility, (b) interference effect of the choline sensor, and (c) the response time.

To analyze the reproducibility performance of the choline bio-sensor, the test was performed at a 0.01 μM concentration of choline and an applied potential of 0 to +1.5 V in the phosphate buffer medium with pH 5.7, as demonstrated in Fig. 5(a). As it is shown in Fig. 5(a), the seven runs are practically indistinguishable in an identical condition. The resulted electrochemical responses did not change even after the washing of the electrode after each trial. Therefore, the result of this reproducibility test provides the evidence for the reliability of the choline sensor.

**Fig. 6:**
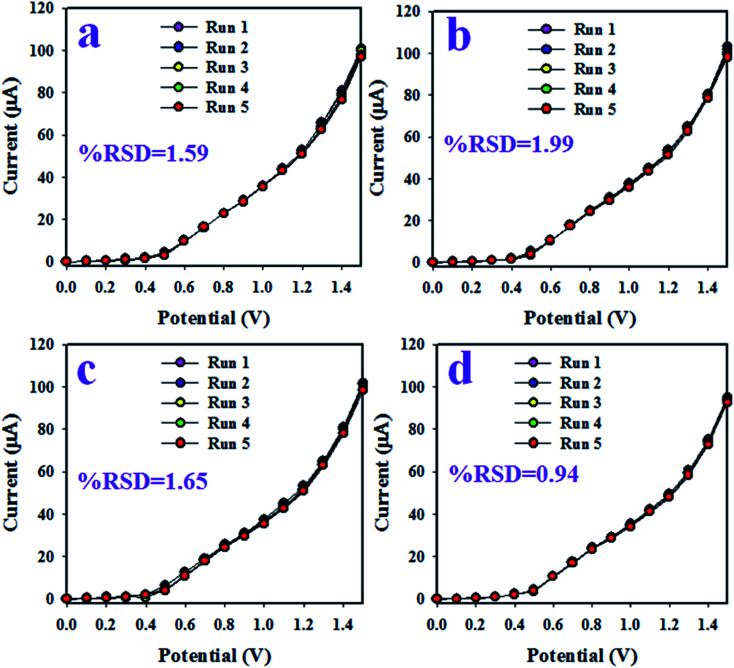
The validation test of the choline sensor based on Mn_2_O_3_/NiO NP/binder/GCE. (a) Reproducibility at 1^st^ day, (b) reproducibility at 2^nd^ day, (c) reproducibility at 3^rd^ day, and (d) reproducibility at 4^th^ day.

The interference effect of the choline sensor was determined and the corresponding data are shown in Fig. 5(b). This test was done with a 0.01 μM concentration of choline and an applied potential of 0 to +1.5 V in an optimized buffer system. The electrochemical (*I*–*V*) responses are not altered in the presence of interfering metal ions, such as Na^+^, K^+^, Mg^2+^ and Ca^2+^. Therefore, the projected choline bio-sensor is free of interference effect during the analysis of biological samples. The response time is an important criterion of a sensor and it provides information about the efficiency of the sensor. This test is performed at a 0.01 μM concentration of choline and an applied potential of 0 to +1.5 V in a phosphate buffer medium. As it is demonstrated in Fig. 5(c), the obtained response time is around 16 s, a result that might be satisfactory. To estimate the accuracy of the reproducibility test, the percentage of the relative standard deviation of current data were measured and it was found to be 0.90%. Thus, it can be concluded that the projected choline bio-sensor was able to perform reliably in real fields of application. Similar reproducibility tests of the choline bio-sensor were performed for an elongated period of four days, as shown in Fig. 6(a–d). The analogous observations are obtained, as shown in Fig. 5(a).

The choline bio-sensor was fabricated by attaching active Mn_2_O_3_/NiO NPs onto a GCE and the binding strength between NPs and GCE was improved by the addition of Nafion (5% Nafion suspension in ethanol). The fabricated sensor was applied to detect choline in the phosphate buffer medium. At the very initial phase, the surface coverage due to the adsorption of a few number of choline molecules on the surface of the working electrode is small, and as a result, the corresponding reaction rate is very slow. With the increase in the surface coverage, the rate of reaction increased and approached a steady state equilibrium condition. Initially, a saturated surface coverage on the working electrode surface occurred and an equilibrium rate of reaction was observed. At this condition, the steady state current density was experimented in the phosphate buffer medium. Such steady state current data are demonstrated in Fig. 4(d). As presented in Fig. 4(d), steady state current data are homogeneously distributed along the linear plot. Thus, this homogeneous linear distribution of current data provide evidence about the reliability of the method. As it is shown in Fig. 5(c), the response time of the choline bio-sensor is 16 s. Therefore, this 16 s is necessary for the choline bio-sensor to achieve the steady state *I*–*V* response. Considering the moderately high sensitivity (16.4557 μA μM^−1^ cm^−2^) of the choline bio-sensor based on Mn_2_O_3_/NiO NPs on GCE, it can be assumed that the projected choline sensor has active catalytic decomposability and high adsorption ability.^47,48^ A comparison of recent research activities on the choline bio-sensor^49–53^ was performed, and the corresponding data based on sensitivity, detection limit (DL) and linear dynamic range (LDR) are represented in Table 1. According to the presented data in Table 1, the choline bio-sensor based on Mn_2_O_3_/NiO NP/binder/GCE shows significant and qualified analytical performances.

**Table 1:** Comparison of the sensor performance with similar work based on different modified electrodes by an electrochemical approach^a^

Modified GCE	DL	LDR	Sensitivity	Ref.
ChOx/PDDA/PB/FePO_4_/GC	0.4 μM	2 μM to 3.2 mM	0.0752 μA μM^−1^ cm^−2^	49
MWCNT/GNp/ChOx/PDDA/Pt^c^	0.3 μM	0.001–0.5 mM	0.183 μA μM^−1^ cm^−2^	50
PVA/Au/ChOx/Pt^f^	10 μM	0.02–0.4 mM	0.0157 μA μM^−1^ cm^−2^	51
ChOx/poly(1,2-DAB) NTs/Pt^k^	50 μM	0.05–2 mM	0.0 67 μA μM^−1^ cm^−2^	52
(ChOx/PDDA)_*n*_/(PVS/PAA)_3_/Pt	0.2 μM	0.005–0.1 mM	0.1774 μA μM^−1^ cm^−2^	53
Mn_2_O_3_/NiO NPs onto GCE	5.77 pM	0.1 nM to 0.1 mM	16.4557 μA μM^−1^ cm^−2^	This study

a DL (detection limit) and LDR (linear dynamic range).

**Scheme 1:**
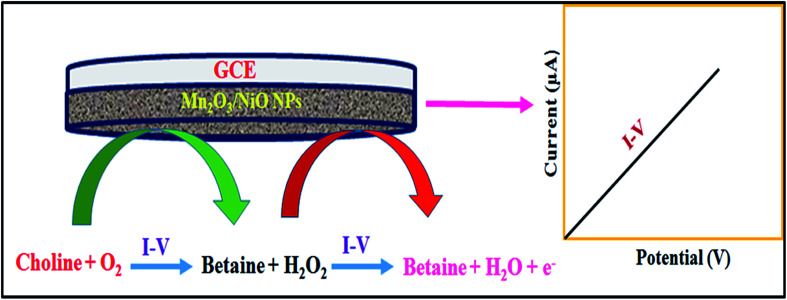
Pictorial presentation of choline detection in the phosphate buffer medium in electrochemical approaches.

In the electrochemical detection process of choline based on the Mn_2_O_3_/NiO NP/binder/GCE in the optimized buffer medium, choline and the dissolved oxygen molecules are adsorbed onto the surface of the working electrode in the electrochemical method (with respect to the applied potential), and betaine and hydrogen peroxide are produced. Subsequently, resulted H_2_O_2_ was automatically converted into H_2_O and released electrons (e^−^). These released electrons significantly enhanced the resultant current in the electrochemical process. Similar electrochemical observations of the choline detection have been reported in previous articles.^54,55^ Therefore, the enrichment of electrons is observed in the sensing medium as well as in the conductivity by *I*–*V* responses. This is the major objective to have the elevated electrochemical response with the increase in the choline concentration, as presented in Fig. 4(c). From this enzyme-less selective choline sensor study, it was observed that the sensor is most selective and highly sensitive towards choline with low-dimensional ternary mixed Mn_2_O_3_/NiO nanoparticles by electrochemical approaches compared to other published non-enzymatic sensors with different sensing matrices (Scheme 1).^56–76^

### Analysis of real samples

To study the validity of the proposed sensor based on Mn_2_O_3_/NiO NP/binder/GCE in real biological samples, such as human serum, mouse serum and rabbit serum, were analyzed. The results are presented in Table 2. As it is demonstrated in Table 2, the electrochemical responses show that it is possible to reliably apply the proposed choline sensor based on Mn_2_O_3_/NiO NP/binder/GCE to real samples.

**Table 2:** Analyses of real biological samples with the Mn_2_O_3_/NiO NP/binder/GCE sensor

Sample	Added choline conc. (μM)	Measured choline conc.^a^ by Mn_2_O_3_/NiO NP/binder/GCE (μM)			Average recovery^b^ (%)	RSD^c^ (%) (*n* = 3)
		R1	R2	R3		
Human serum	0.01	0.009901	0.009885	0.009835	98.74	0.35
Mouse serum	0.01	0.009732	0.009637	0.009585	96.51	0.77
Rabbit serum	0.01	0.009973	0.009942	0.009954	99.56	0.16

a Mean of three repeated determination (signal to noise ratio 3) with Mn_2_O_3_/NiO NPs/binder/GCE.

b Concentration of choline determined/Concentration taken (unit: nM).

c Relative standard deviation value indicates precision among three repeated measurements (R1, R2, R3).

## Conclusion

A choline sensor with Mn_2_O_3_/NiO NPs was fabricated *via* a wet-chemical (co-precipitation process) process at a low temperature. The as-synthesized NPs were fully characterized *via* powder X-ray diffraction, XPS, UV-Vis, FTIR, FESEM and EDS analyses. A thin layer of NPs was deposited onto the GCE with a conducting Nafion binder to result in the working electrode of the desire choline sensor. A calibration curve was plotted as current *versus* concentration of choline in this approach. The sensitivity (16.4557 μA μM^−1^ cm^−2^) and detection limit (5.77 ± 0.29 pM) were calculated from the slope of the calibration curve. The proposed sensor was found to be linear over the LDR (0.1 nM to 0.1 mM) of choline at an applied potential of +1.5 V. This novel research approach might be a reliable way to develop future sensors in the field of biomedical sector in broad scales.

## Conflicts of interest

There are no conflicts to declare.

## Supplementary Material

## References

[cit1] Institute of Medicine , Dietary reference intakes for thiamin, riboflavin, niacin, vitamin B6, folate, vitamin B12, pantothenic acid, biotin, and choline, National Academy Press, Washington, DC, USA, 1998, pp. 390–422 23193625

[cit2] Zeisel S. H., Mar M. H., Howe J. C., Holden J. M. (2003). Concentrations of choline-containing compounds and betaine in common foods. J. Nutr..

[cit3] Hussain M. A., Juergen G., Rima O. (2012). The role of choline in prostate cancer. Clin. Biochem..

[cit4] Zeisel S. H., da Costa K. A. (2009). Choline: an essential nutrient for public health. Nutr. Rev..

[cit5] Zeisel S. H. (2006). The fetal origins of memory: the role of dietary choline in optimal brain development. J. Pediatr..

[cit6] Zeisel S. H., Blusztajn J. K. (1994). Choline and human nutrition. Annu. Rev. Nutr..

[cit7] Buchman A. L., Dubin M. D., Moukarzel A. A., Jenden D. J., Roch M., Rice K. M., Gornbein J., Ament M. E. (1995). Choline deficiency: a cause of hepatic steatosis during parenteral nutrition that can be reversed with intravenous choline supplementation. Hepatology.

[cit8] Zeisel S. H., Da Costa K. A., Franklin P. D., Alexander E. A., Lamont J. T., Sheard N. F., Choline A. B. (1991). an essential nutrient for humans. FASEB J..

[cit9] Albright C. D., Liu R., Bethea T. C., Da Costa K. A., Salganik R. I., Zeisel S. H. (1996). Choline deficiency induces apoptosis in SV40-immortalized CWSV-1 rat hepatocytes in culture. FASEB J..

[cit10] da Costa K. A., Gaffney C. E., Fischer L. M., Zeisel S. H. (2005). Choline deficiency in mice and humans is associated with increased plasma homocysteine concentration after a methionine load. Am. J. Clin. Nutr..

[cit11] Zeisel S. H. (2000). Choline: an essential nutrient for humans. Nutrition.

[cit12] Pal S., Sharma M. K., Danielsson B., Willander M., Chatterjee R., Bhand S. (2014). A miniaturized nanobiosensor for choline analysis. Biosens. Bioelectron..

[cit13] Deshpande K., Danielsson B., Bhand S. (2011). Flow injection analysis of choline in milk and dietary supplements using an enzyme thermistor. Chem. Senses.

[cit14] Takayama M., Itoh S., Nagasaki T., Tanimizu I. (1977). A new enzymatic method for determination of serum choline-containing phospholipids. Clin. Chim. Acta.

[cit15] Chen Z., Ren X., Meng X., Chen D., Yan C., Ren J., Yuan Y., Tang F. (2011). Optical detection of choline and acetylcholine based on H_2_O_2_-sensitive quantum dots. Biosens. Bioelectron..

[cit16] Qin X., Wang H., Wang X., Miao Z., Chen L., Zhao W., Shan M., Chen Q. (2011). Sens. Actuators, B.

[cit17] Bhat S. V., Deepak F. L. (2005). Tuning the bandgap of ZnO by substitution with Mn^2+^, Co^2+^ and Ni^2+^. Solid State Commun..

[cit18] Tompsett D. A., Middlemiss D. S., Islam M. S. (2012). Importance of anisotropic Coulomb interactions and exchange to the band gap and antiferromagnetism of β-MnO_2_ from DFT + U. Phys. Rev. B: Condens. Matter Mater. Phys..

[cit19] Towler M. D., Allan N. L. (1994). Ab initio study of Mno and Nio. Phys. Rev. B: Condens. Matter Mater. Phys..

[cit20] Rahman M. M., Alam M. M., Asiri A. M. (2017). Fabrication of acetone sensor based on facile ternary MnO_2_/Gd_2_O_3_/SnO_2_ nanosheets for environmental safety. New J. Chem..

[cit21] Rahmana M. M., Alam M. M., Asiri A. M., Islam M. A. (2018). 3,4-Diaminotoluene sensor development based on hydrothermally prepared MnCo_x_O_y_ nanoparticles. Talanta.

[cit22] Rahman M. M., Marwani H. M., Algethami F. K., Asiri A. M. (2017). Comparative performance of hydrazine sensors developed with Mn_3_O_4_/carbon-nanotubes, Mn_3_O_4_/graphene-oxides and Mn_3_O_4_/carbon black nanocomposites. Mater. Express.

[cit23] Subhan M. A., Saha P. C., Rahman M. M., Ahmed J., Asiri A. M., Al-Mamun M. (2018). Fabrication of a 2,4-dinitrophenol sensor based on Fe_3_O_4_@Ag@Ni nanomaterials and studies on their antibacterial properties. New J. Chem..

[cit24] Hussain M. M., Rahman M. M., Asiri A. M. (2017). Ultrasensitive and selective 4-aminophenol chemical sensor development based on nickel oxide nanoparticles decorated carbon nanotube nanocomposites for green environment. J. Environ. Sci..

[cit25] Rahman M. M., Alam M. M., Asiri A. M. (2018). Sensitive 1,2-dichlorobenzene chemi-sensor development based on solvothermally prepared FeO/CdO nanocubes for environmental safety. J. Ind. Eng. Chem..

[cit26] Rahman M. M., Alam M. M., Asiri A. M. (2018). 2-Nitrophenol sensor-based wet-chemically prepared binary doped Co_3_O_4_/Al_2_O_3_ nanosheets by an electrochemical approach. RSC Adv..

[cit27] Rahman M. M., Alam M. M., Asiri A. M., Awual M. R. (2017). Fabrication of 4-aminophenol sensor based on hydrothermally prepared ZnO/Yb_2_O_3_ nanosheets. New J. Chem..

[cit28] Zhao Y., Jiang P., Xie S. S. (2013). ZnO-template-mediated synthesis of three-dimensional coral-like MnO_2_ nanostructure for supercapacitors. J. Power Sources.

[cit29] Reddy A. L. M., Shaijumon M. M., Gowda S. R., Ajayan P. M. (2009). Coaxial MnO_2_/carbon nanotube array electrodes for high-performance lithium batteries. Nano Lett..

[cit30] Huang M., Zhang Y., Li F., Wang Z., Alamusi, Hu N., Wen Z., Liu Q. (2014). Merging of Kirkendall Growth and Ostwald Ripening: CuO@MnO_2_ Core-shell Architectures for Asymmetric Supercapacitors. Sci. Rep..

[cit31] Xiao X., Zhang F., Feng Z., Deng S., Wang Y. (2015). Adsorptive removal and kinetics of methylene blue from aqueous solution using NiO/MCM-41 composite. Phys. E.

[cit32] Liu L., Zhang H., Yang J., Mu Y., Wang Y. (2015). Self-assembled novel dandelion-like NiCo_2_O_4_ microspheres@nanomeshes with superior electrochemical performance for supercapacitors and lithium-ion batteries. J. Mater. Chem. A.

[cit33] Liu Z. Q., Chen G. F., Zhou P. L., Li N., Su Y. Z. (2016). Building layered NixCo_2x_(OH)_6x_ nanosheets decorated three dimensional Ni frameworks for electrochemical applications. J. Power Sources.

[cit34] Kalaiyarasan G., Aswathi K., Joseph J. (2017). Formation of nanoporous NiS films from electrochemically modified GC surface with Nickel Hexacyanoferrate film and its performance for the hydrogen evolution reaction. Int. J. Hydrogen Energy.

[cit35] Cui Y., Wang C., Wu S., Liu G., Zhang F., Wang T. (2011). Lotus-root-like NiO nanosheets and flower-like NiO microspheres: synthesis and magnetic properties. CrystEngComm.

[cit36] Mohan S., Srivastava P., Maheshwari S. N., Sundar S., Prakash R. (2011). Nano-structured nickel oxide based DNA biosensor for detection of visceral leishmaniasis (Kala-azar). Analyst.

[cit37] Zhang W., Yang Z., Liu Y., Tang S., Han X., Chen M. (2004). Controlled synthesis of Mn_3_O_4_ nanocrystallites and MnOOH nanorods by a solvothermal method. J. Cryst. Growth.

[cit38] Khalaji A. D., Das D. (2014). Synthesis and characterizations of NiO nanoparticles *via* solid-state thermal decomposition of nickel(II) Schiff base complexes. Int. Nano Lett..

[cit39] Sharrouf M., Awad R., Roumié M., Marhaba S. (2015). Structural, Optical and Room Temperature Magnetic Study of Mn_2_O_3_ Nanoparticles. Mater. Sci. Appl..

[cit40] Niu X., Wei H., Tang K., Liu W., Zhao G., Yang Y. (2015). Solvothermal synthesis of 1D nanostructured Mn_2_O_3_: effect of Ni^2+^ and Co^2+^ substitution on the catalytic activity of nanowires. RSC Adv..

[cit41] Yan H., Zhang D., Xu J., Lu Y., Liu Y., Qiu K., Zhang Y., Luo Y. (2014). Solution growth of NiO nanosheets supported on Ni foam as high-performance electrodes for supercapacitors. Nanoscale Res. Lett..

[cit42] Abbasi M. A., Ibupoto Z. H., Hussain M., Khan Y., Khan A., Nur O., Willander M. (2012). Potentiometric Zinc Ion Sensor Based on Honeycomb-Like NiO Nanostructures. Sensors.

[cit43] Subhan M. A., Saha P. C., Alam M. M., Asiri A. M., Al-Mamund M., Rahman M. M. (2018). Development of Bis-Phenol A sensor based on Fe2MoO4·Fe3O4·ZnO nanoparticles for sustainable environment. J. Environ. Chem. Eng..

[cit44] Rahman M. M., Alam M. M., Asiri A. M. (2018). 2-Nitrophenol sensor-based wet-chemically prepared binary doped Co_3_O_4_/Al_2_O_3_ nanosheets by an electrochemical approach. RSC Adv..

[cit45] Rahman M. M., Alam M. M., Asiri A. M., Awual M. R. (2017). Fabrication of 4-aminophenol sensor based on hydrothermally prepared ZnO/Yb_2_O_3_ nanosheets. New J. Chem..

[cit46] Rahman M. M., Alam M. M., Asiri A. M., Islam M. A. (2017). Fabrication of selective chemical sensor with ternary ZnO/SnO_2_/Yb_2_O_3_ nanoparticles. Talanta.

[cit47] Khan S. B., Faisal M., Rahman M. M., Jamal A. (2011). Low-temperature growth of ZnO nanoparticles: Photocatalyst and acetone sensor. Talanta.

[cit48] Umar A., Rahman M. M., Hahn Y. B. (2009). Ultra-sensitive hydrazine chemical sensor based on high-aspect-ratio ZnO nanowires. Talanta.

[cit49] Zhang H., Yin Y., Wu P., Cai C. (2012). Indirect electrocatalytic determination of choline by monitoring hydrogen peroxide at the choline oxidase-prussian blue modified iron phosphate nanostructures. Biosens. Bioelectron..

[cit50] Qin X., Wang H., Wang X., Miao Z., Chen L., Zhao W., Shan M., Chen Q. (2010). Amperometric biosensors based on gold nanoparticles-decorated multiwalled carbon nanotubes-poly(diallyldimethylammonium chloride) biocomposite for the determination of choline. Sens. Actuators, B.

[cit51] Ren X., Tang F., Liao R., Zhang L. (2009). Using gold nanorods to enhance the current response of a choline biosensor. Electrochim. Acta.

[cit52] Curulli A., Valentini F., Orlanduci S., Terranova M. L., Palleschi G. (2004). Pt based enzyme electrode probes assembled with Prussian Blue and conducting polymer nanostructures. Biosens. Bioelectron..

[cit53] Qin X., Wang H., Wang X., Li S., Miao Z., Huang N., Chen Q. (2009). Amperometric choline biosensors based on multi-wall carbon nanotubes and layer-by-layer assembly of multilayer films composed of Poly(diallyldimethylammonium chloride) and choline oxidase. Mater. Sci. Eng., C.

[cit54] Thiagarajan V., Madhurantakam S., Sethuraman S., Rayappan J. B. B., Krishnan U. M. (2016). Nano interfaced biosensor for detection of choline in triple negative breast cancer cells. J. Colloid Interface Sci..

[cit55] Mansor N. N. N., Leong T. T., Safitri E., Futra D., Ahmad N. S., Nasuruddin D. N., Itnin A., Zaini I. Z., Arifin K. T., Heng L. Y., Hassan N. I. (2018). An Amperometric Biosensor for the Determination of Bacterial Sepsis Biomarker, Secretory Phospholipase Group 2-IIA Using a Tri-Enzyme System. Sensors.

[cit56] Ahmad R., Wolfbeis O. S., Hahn Y. B., Alshareef H. N., Torsi L., Salama K. N. (2018). Deposition of nanomaterials: A crucial step in biosensor fabrication. Mater. Today Commun..

[cit57] Awual M. R., Hasan M. M., Islam A., Rahman M. M., Asiri A. M., Khaleque M. A., Sheikh M. C. (2019). Offering an innovative composited material for effective lead(II) monitoring and removal from polluted water. J. Cleaner Prod..

[cit58] Alam M. M., Asiri A. M., Uddin M. T., Islam M. A., Awual M. R., Rahman M. M. (2019). Detection of uric acid based on doped ZnO/Ag2O/Co3O4 nanoparticles fabricated glassy carbon electrode. New J. Chem..

[cit59] Awual M. R., Hasan M. M., Eldesoky G. E., Khaleque M. A., Rahman M. M., Naushad M. (2016). Facile mercury detection and removal from aqueous media involving ligand impregnated conjugate nanomaterials. Chem. Eng. J..

[cit60] Hussain M. M., Rahman M. M., Asiri A. M., Awual M. R. (2016). Non-enzymatic simultaneous detection of L-glutamic acid and uric acid using mesoporous Co_3_O_4_ nanosheets. RSC Adv..

[cit61] Awual M. R., Islam A., Hasan M. M., Rahman M. M., Asiri A. M., Khaleque M. A., Sheikh M. C. (2019). Introducing an alternate conjugated material for enhanced lead(II) capturing from wastewater. J. Cleaner Prod..

[cit62] Arshad M. N., Sheikh T. A., Rahman M. M., Asiri A. M., Marwany H. M., Awual M. R. (2017). Fabrication of cadmium ionic sensor based on (E)-4-Methyl-N'-(1-(pyridin-2-yl)ethylidene) benzenesulfonohydrazide (MPEBSH) by electrochemical approach. J. Organomet. Chem..

[cit63] Awual M. R., Alharthi N. H., Okamoto Y., Karim M. R., Halim M. E., Hasan M. M., Rahman M. M., Islam M. M. (2017). Ligand field effect for Dysprosium(III) and Lutetium(III) adsorption and EXAFS coordination with novel composite nanomaterials. Chem. Eng. J..

[cit64] Rahman M. M., Alam M. M., Asiri A. M., Awual M. R. (2017). Fabrication of 4-aminophenol sensor based on hydrothermally prepared ZnO/Yb_2_O_3_ nanosheets. New J. Chem..

[cit65] Awual M. R., Alharthi N. H., Hasan M. M., Karim M. R., Islam A., Znad H., Hossain M. A., Halim M., Rahman M. M., Khaleque M. A. (2017). Inorganic-organic based novel nano-conjugate material for effective cobalt(II) ions capturing from wastewater. Chem. Eng. J..

[cit66] Sheikh T. A., Arshad M. N., Rahman M. M., Asiri A. M., Marwani H. M., Awual M. R., Bawazir W. A. (2017). Trace electrochemical detection of Ni^2+^ ions with bidentate N,N'-(ethane-1,2-diyl)bis(3,4-dimethoxybenzenesulfonamide)
[EDBDMBS] as a chelating agent. Inorg. Chim. Acta.

[cit67] Awual M. R., Khraisheh M., Alharthi N. H., Luqman M., Islam A., Karim M. R., Rahman M. M., Khaleque M. A. (2018). Efficient detection and adsorption of cadmium(II) ions using innovative nano-composite materials. Chem. Eng. J..

[cit68] Sheikh T. A., Rahman M. M., Asiri A. M., Marwani H. M., Awual M. R. (2018). 4-Hexylresorcinol sensor development based on wet-chemically prepared Co_3_O_4_@Er2O3 nanorods: A practical approach. J. Ind. Eng. Chem..

[cit69] Awual M. R., Hasan M. M., Rahman M. M., Asiri A. M. (2019). Novel composite material for selective copper(II) detection and removal from aqueous media. J. Mol. Liq..

[cit70] Alam M. M., Asiri A. M., Uddin M. T., Inamuddin M. A. I., Awual M. R., Rahman M. M. (2019). One-step wet-chemical synthesis of ternary ZnO/CuO/Co3O4 nanoparticles for sensitive and selective melamine sensor development. New J. Chem..

[cit71] Awual M. R., Asiri A. M., Rahman M. M., Alharthi N. H. (2019). Assessment of enhanced nitrite removal and monitoring using ligand modified stable conjugate materials. Chem. Eng. J..

[cit72] Rahman M. M., Sheikh T. A., Asiri A. M., Awual M. R. (2019). Development of 3-methoxyanaline sensor probe based on thin Ag_2_O@La_2_O_3_ nanosheets for environmental safety. New J. Chem..

[cit73] Awual M. R., Hasan M. M., Asiri A. M., Rahman M. M. (2019). Novel optical composite material for efficient vanadium(III) capturing from wastewater. J. Mol. Liq..

[cit74] Rahman M. M., Hussain M. M., Arshad M. N., Awual M. R., Asiri A. M. (2019). Arsenic sensor development based on modified with (E)-N'-(2-Nitrobenzylidine)-benzenesulfonohydrazide: A real sample analysis. New J. Chem..

[cit75] Awual M. R., Hasan M. M., Islam A., Rahman M. M., Asiri A. M., Khaleque M. A., Sheikh M. C. (2019). Introducing an amine functionalized novel conjugate material for toxic nitrite detection and adsorption from wastewater. J. Cleaner Prod..

[cit76] Awual M. R., Hasan M., Asiri A. M., Rahman M. M. (2019). Cleaning the arsenic(V) contaminated water for safe-guarding the public health using novel composite material. Composites, Part B.

